# Age- and Treatment-Related Patterns in Fatigue, Coping/Resilience, and Skeletal Muscle Bioenergetics (^31^P-MRS τPCr) in Cancer Survivors: Exploratory Pilot Analysis

**DOI:** 10.3390/biomedicines14020448

**Published:** 2026-02-17

**Authors:** Nada Lukkahatai, Susan Grayson, Michael A. Carducci, Christopher M. Bergeron, Kenneth W. Fishbein, Richard G. Spencer, Leorey N. Saligan

**Affiliations:** 1 School of Nursing, Johns Hopkins University, Baltimore, MD 21205, USA; 2 Marcella Niehoff School of Nursing, Loyola University Chicago, Chicago, IL 60660, USA; sgrayson@luc.edu; 3 School of Medicine, Johns Hopkins University, Baltimore, MD 21205, USA; carducci@jhmi.edu; 4 Laboratory of Clinical Investigation, Intramural Research Program, National Institute on Aging, National Institutes of Health, Baltimore, MD 21224, USAspencerri@mail.nih.gov (R.G.S.); 5 School of Nursing, Rutgers, The State University of New Jersey, Newark, NJ 07102, USA; ls1684@sn.rutgers.edu

**Keywords:** cancer-related symptoms, physical function, mitochondrial energetics, resilience, fatigue, aging

## Abstract

**Background**: Cancer-related health outcomes are shaped by the interplay of aging, complex treatment exposures, and individual psychological characteristics. Mitochondrial dysfunction has been implicated as an underlying biological process affecting cancer-related outcomes. This secondary, exploratory pilot analysis aimed to examine age- and treatment-related differences in fatigue, coping self-efficacy, resilience, and skeletal muscle mitochondrial oxidative capacity, measured via phosphorus-31 magnetic resonance spectroscopy (^31^P-MRS). **Methods**: Eleven cancer survivors (mean age 53.3 ± 12.7 years) were recruited from a larger symptom management trial. Participants underwent ^31^P-MRS to assess mitochondrial function via phosphocreatine recovery time constant (τPCr). Patient-reported outcome measures and physical function assessments were collected. Group comparisons and correlation analyses were conducted to evaluate differences and associations based on age (<65 vs. ≥65 years) and treatment. Because treatment categories were not mutually exclusive and the time since last treatment was not collected, treatment-related comparisons are descriptive only. Given the small available sample size, we conducted this study as exploratory and hypothesis-generating. **Results**: Older survivors (≥65) had longer τPCr (59.5 vs. 50.1 s), weaker grip strength, higher fatigue, and lower physical performance compared to younger participants, although differences were not statistically significant. Treatment-related patterns were descriptive; participants receiving multiple treatments had shorter τPCr but lower muscular strength, while immunotherapy recipients reported higher fatigue and lower physical activity. Among younger participants, a negative correlation was observed between τPCr and fatigue (ρ = −0.71), and positive correlations were observed with resilience (ρ = 0.61) and coping self-efficacy (ρ = 0.74), reflecting a pattern that warrants cautious interpretation in this small sample. **Conclusions**: These preliminary results suggest age- and treatment-related differences in fatigue, physical performance, psychological factors, and skeletal muscle mitochondrial bioenergetics. These signals warrant further testing in larger, adequately powered cohorts to clarify mechanisms and inform the development of personalized survivorship care strategies.

## 1. Introduction

Cancer-related health outcomes are influenced by a complex interplay of biological aging, cumulative treatment exposures, and psychological factors such as coping and resilience [1,2] Aging is associated with physiological changes, including reduced regenerative capacity, increased systemic inflammation, and oxidative stress, that may amplify vulnerability to cancer treatment-related toxicities [3]. Concurrently, cancer treatments such as chemotherapy and radiation contribute to off-target cellular injury, leading to persistent symptoms such as fatigue, weakness, and cognitive decline [4,5,6,7]. These effects are particularly pronounced in older adults, who often experience diminished physiological reserve and impaired cellular function, including mitochondrial health [4,8].

Mitochondrial dysfunction has emerged as a central biological mechanism linking aging and cancer treatment-related toxicities. Mitochondria are essential for ATP production, redox regulation, and cellular homeostasis, but are especially vulnerable to damage from both intrinsic aging processes and extrinsic cancer therapies. Chemotherapy and radiation can directly impair mitochondrial DNA, disrupt electron transport chain function, and promote the accumulation of reactive oxygen species (ROS), triggering oxidative stress and cellular instability [9,10]. Independently, aging also contributes to mitochondrial decline, including impaired oxidative phosphorylation, elevated ROS, and increased pro-inflammatory signaling, which has been implicated in frailty, sarcopenia, and neurodegeneration [4,11,12]. When combined, the biological stressors of aging and cancer therapy may accelerate mitochondrial dysfunction, contributing to a chronic, energetically inefficient state that underlies persistent symptoms and functional decline in cancer survivors [6,13,14,15]. Given the nonspecific nature of many cancer therapies, widespread mitochondrial and systemic complications often arise, negatively impacting physical and psychological recovery [16,17]. These plausible mechanistic links between mitochondrial dysfunction and fatigue warrant further investigation, of which this exploratory secondary analysis is the first step in generating hypotheses and establishing feasibility.

The role of mitochondrial dysfunction in cancer survivorship is increasingly recognized. However, very few studies have used in vivo ^31^P-MRS to directly assess skeletal muscle oxidative capacity in this population [18]. Even fewer have examined how MRS-derived measures such as the phosphocreatine recovery time constant (τPCr) relate to psychological outcomes like resilience and coping. Prior literature has indicated that resilience and coping may affect psychobiological adaptation and stress responses in older adults, including through hypothalamus–pituitary–adrenal axis function and diurnal cortisol regulation [19,20]. The novel incorporation of mitochondrial dysfunction data with resilience and coping in this study aims to add a new facet to the literature examining psychobiological adaptation in chronic disease populations.

Systematic reviews have characterized τPCr across health and disease broadly [21], and earlier work in non-cancer populations shows τPCr correlates well with biopsy-derived mitochondrial oxidative capacity [22]. ^31^P-MRS provides a noninvasive biomarker of mitochondrial function through τPCr, which reflects the muscle’s ability to resynthesize ATP via oxidative phosphorylation [23]. By pairing τPCr with patient-reported outcomes and functional measures, this study uses novel measures to extend the literature to capture both biological energetics and psychosocial dimensions of survivorship.

This study is a secondary, exploratory analysis of data from a subset of participants in a registered intervention trial and is not powered to detect statistical interactions. This analysis differs from the earlier publication [18], which focused broadly on associations between mitochondrial capacity and function in cancer survivors. The present report extends that work by:

- Conducting age-stratified comparisons (<65 vs. ≥65 years);
- Describing treatment-related patterns across overlapping modalities;
- Including additional fatigue and fatigability measures (e.g., FACIT-F) alongside psychological constructs.

Instead, our purpose is to understand how patient-reported outcomes (PROs), muscle strength, physical activity, and mitochondrial energetics in skeletal muscle, as measured by the τPCr using ^31^P-MRS, vary across age groups and cancer treatment modality. Specifically, we examined (1) how PROs, muscle strength, physical activity, psychological characteristics (i.e., coping self-efficacy, resilience), and skeletal muscle mitochondrial oxidative capacity differ between older (≥65 years) and younger (<65 years) cancer survivors, and (2) how these variables differ by cancer treatment modality.

Based on prior literature, we anticipated that τPCr might relate to fatigue, coping self-efficacy, resilience, and age; however, these analyses were exploratory and hypothesis-generating. By investigating these relationships, this study seeks to generate hypotheses, provide effect size estimates, and guide future research on how physical and psychological factors, as well as disruptions in cellular energy metabolism, shape cancer health outcomes across aging and treatment exposures. This supports the long-term goal of guiding the development of targeted interventions to improve survivorship trajectories.

## 2. Materials and Methods

### 2.1. Sample Recruitment

This is a secondary, exploratory analysis of data originally collected for a study examining the associations between skeletal muscle mitochondrial oxidative capacity and physical and psychosocial outcomes in cancer survivors [18]. This cross-sectional analysis was designed and reported according to STROBE reporting guidelines. Participants were recruited from oncology outpatient clinics affiliated with a university hospital and initially enrolled in a parent study examining the effects of a home exercise program on well-being in cancer survivors. The parent study was registered at ClinicalTrials.gov (NCT03576274).

Eligibility criteria for the exploratory MRS sub-study included the absence of contraindications to MRI/MRS (e.g., metal implants, claustrophobia), no recent history of substance use disorder, and not being pregnant or lactating at the time of screening. Participation in the ^31^P-MRS sub-study was optional and dependent on MRI eligibility, scanner availability, and participant willingness. A total of 11 cancer survivors participated in the ^31^P-MRS sub-study between September 2020 and February 2022 [18].

Participation in the ^31^P-MRS sub-study was optional and dependent on MRI eligibility, scanner availability, and participant willingness. As such, participants who completed the imaging protocol may represent a selective subgroup of cancer survivors, potentially those who were more physically active, health-conscious, or more willing to engage in additional study procedures. Prior research suggests that individuals who volunteer for imaging studies often differ systematically from those who do not [24]. Because systematic documentation of the number of individuals approached, screened, or declining participation was not maintained within the parent trial, the proportion of eligible participants who completed the MRS protocol cannot be determined. This limits the ability to quantify potential selection bias and may affect the generalizability of findings from this small exploratory sample.

### 2.2. Measures

#### 2.2.1. Patient-Reported Outcomes

Patient-reported outcomes were assessed using validated instruments to capture the multidimensional experience of cancer-related symptoms, psychological well-being, and functional status. As previously reported in Gonsalves et al. (2024), these measures included the PROMIS^®^-29 Profile v2.0, which assesses key health domains such as fatigue, anxiety, depression, sleep disturbance, physical function, pain, and participation in social roles, as well as the 36-Item Short Form Health Survey (SF-36) to evaluate physical and mental health quality of life [18]. In this analysis, we included the PROMIS^®^ fatigue subscale to measure fatigue. The Connor-Davidson Resilience Scale (CD-RISC) and the Coping Self-Efficacy Scale (CSE-13) were also used to assess psychological resilience and coping strategies. Sociodemographic and clinical data, including age, sex, cancer diagnosis, treatment modality, and time since diagnosis, were collected by self-report.

To deepen fatigue assessment beyond global symptom reporting, this study also included the Functional Assessment of Chronic Illness Therapy–Fatigue (FACIT-F) scale [25]. The FACIT-F was selected for its specificity in measuring how fatigue interferes with daily activities and its broad validation in oncology populations. Furthermore, the Pittsburgh Fatigability Scale was used to separately evaluate physical and mental fatigability in response to hypothetical tasks. Unlike PROMIS fatigue scores, which reflect general symptom severity, fatigability measures provide insights into the individual’s perceived capacity to perform physical and cognitive tasks, which is particularly relevant for understanding the functional limitations accompanying mitochondrial dysfunction. Fatigue was therefore assessed using complementary instruments to capture related but distinct constructs. PROMIS-Fatigue primarily reflects global perceived fatigue severity, FACIT-F emphasizes fatigue-related interference with daily functioning, and the Pittsburgh Fatigability Scale captures context-specific fatigue responses to standardized activities (i.e., fatigability). Because global fatigue and fatigability represent different dimensions of symptom experience, each measure was analyzed and interpreted as a separate outcome. Divergent findings across instruments should be understood as reflecting differences in construct measurement rather than inconsistency in results.

#### 2.2.2. Physical Performance

Objective physical function was evaluated using multiple standardized tools, as previously reported in Gonsalves et al. (2024) [18]. These included the Short Physical Performance Battery (SPPB), comprising gait speed, chair stands, and balance tasks, and bilateral handgrip strength, assessed using a dynamometer to evaluate upper-body muscular strength. All assessments were conducted in a standardized clinical setting prior to the ^31^P-MRS procedure to ensure accurate baseline performance metrics.

To evaluate habitual physical activity, participants were instructed to wear a commercially available wearable activity tracker (Fitbit Charge, Fitbit, Inc., San Francisco, CA, USA) throughout the day for seven days following the MRS scan. This timing ensured that activity monitoring did not influence pre-scan recovery state or intramuscular bioenergetic measures. Daily step counts were averaged to estimate free-living mobility and functional engagement in the participants’ natural environment. No formal wear-time validation criteria (e.g., minimum daily hours or number of valid days) were imposed beyond participant instruction to wear the device continuously, and activity data were used descriptively in this exploratory secondary analysis. This approach was taken because physical activity was included as a secondary, descriptive indicator of free-living mobility rather than a primary analytic outcome. As such, step count data were intended to provide contextual information on habitual activity patterns rather than precise estimates of physical activity exposure. Consumer-grade activity trackers have demonstrated acceptable validity and reliability for step count measurement in both healthy adults and clinical populations, including cancer survivors [26,27].

#### 2.2.3. Skeletal Muscle Cellular Energetics

Skeletal muscle oxidative capacity was assessed using ^31^P-MRS to quantify post-exercise phosphocreatine (PCr) recovery to estimate mitochondrial oxidative function. In this study, the τPCr served as the primary outcome measure of mitochondrial capacity. Scans were performed on a 3T Philips Achieva MR scanner (Philips Healthcare, Best, The Netherlands) using a 10-cm ^31^P-tuned transmit-receive surface coil (PulseTeq, Surrey, UK) positioned over the left vastus lateralis muscle. Participants performed a standardized ballistic knee extension exercise while lying supine in the scanner. Spectra were acquired before, during, and after exercise with a temporal resolution of 6 s over a total scan duration of 7.5 min. Exercise duration was calibrated to achieve 33–66% PCr depletion while maintaining intramuscular pH above 6.80 to avoid acidosis.

The recovery of PCr post-exercise was modeled using a mono-exponential function to compute τPCr, which reflects the muscle’s ability to resynthesize ATP via oxidative phosphorylation. Spectral data were processed using the Java-Based Magnetic Resonance User Interface (JMRUI) software (version 5.0; MRUI Consortium, Leuven, Belgium) with non-linear least squares fitting. This validated approach provides an in vivo biomarker of skeletal muscle mitochondrial function and has been used in studies of aging, chronic illness, and functional decline [23]. The complete acquisition protocol, including participant positioning, coil setup, exercise task parameters, spectral resolution, and analytic modeling procedures, has been described in detail in the previous publication [18].

### 2.3. Statistical Analysis

Given the exploratory nature of this study and the small sample size (N = 11), analyses were designed to identify preliminary patterns and generate hypotheses for future research rather than to draw confirmatory conclusions. There were no missing data in this small sample. Descriptive statistics were used to summarize demographic and clinical characteristics. Independent samples (unpaired) *t*-tests were conducted to examine group differences in continuous variables such as τPCr, handgrip strength, and physical function outcomes based on age group (<65 vs. ≥65 years). Descriptive statistics were generated for τPCr, physical function outcomes, and patient-reported outcomes based on treatment modality. To evaluate associations between mitochondrial oxidative capacity and both symptom burden and functional status, Spearman’s rank-order correlations were calculated between τPCr and patient-reported outcomes (PROMIS fatigue, resilience, and coping self-efficacy), as well as objective measures including SPPB scores, handgrip strength, and average daily step count. Correlation analyses were performed in the full sample and stratified by age to explore potential age-related trends and generate effect size estimates for future research. All statistical tests were two-tailed with a significance threshold set at *p* < 0.05. Due to the small sample size (N = 11), bootstrap confidence intervals were not computed, as resampling-based estimates are unstable and may be misleading in very small samples. Accordingly, results are presented descriptively with effect sizes to inform hypothesis generation for future studies. Given the aim of this study to identify promising targets for future research, a false discovery rate was not used. Due to this and the small sample size, any findings, even those of formal statistical significance, should be treated as hypothesis-generating only.

## 3. Results

### 3.1. Sample Description

This analysis included 11 cancer survivors aged 34 to 70 years (M = 53.3, SD = 12.7), drawn from a previously published study (Gonsalves et al., 2024) [18]. The majority were female (54.5%) and identified as White (72.7%). Breast cancer was the most common diagnosis, with participants having received a range of treatments including surgery, chemotherapy, immunotherapy, radiation, and hormone therapy. Most individuals underwent multiple treatment modalities. The average time since cancer diagnosis was approximately six years, although the time since last treatment was not collected in the parent study. The mean τPCr for participants was 52.62 s (SD = 16.31), showing no significant sex-based difference.

### 3.2. Results Stratified by Age

When stratified by age, eight participants (72.7%) were under 65 years old, and three (27.3%) were aged 65 years or older. Age was dichotomized as <65 vs. ≥65 years, consistent with commonly used thresholds in geriatric oncology research. Cohen’s d effect sizes were calculated for age-stratified comparisons to help interpret the magnitude; however, given the very small subgroup size (≥65 years, *n* = 3), these estimates should be interpreted cautiously and are provided for descriptive and future power estimation purposes only.

The mean τPCr was 50.06 s (SD = 16.55) in participants younger than 65 years and 59.49 s (SD = 16.54) for those aged 65 and older. Older adults exhibited longer phosphocreatine recovery times (τPCr = 59.49 ± 16.54 s) compared to younger participants (50.06 ± 16.55 s), indicating reduced skeletal muscle mitochondrial function, although this difference was not statistically significant (*p* = 0.307). Older participants also reported higher self-reported fatigue on the PROMIS-F scale (55.77 ± 8.56 vs. 50.03 ± 12.90) and lower FACIT-F scores (102.22 ± 27.64 vs. 115.22 ± 31.71), both reflecting greater fatigue severity, though differences were not statistically significant.

For physical performance outcomes, older participants had lower SPPB scores (9.67 ± 2.08 vs. 10.75 ± 2.05; *p*= 0.503) and reported lower levels of mental fatigability (7.00 ± 5.66 vs. 17.88 ± 13.07; *p*= 0.400), although these trends were not significant. Older participants also had weaker handgrip strength in both hands, with a significant difference observed for the right hand (36.43 ± 6.86 vs. 56.29 ± 13.19; *p* = 0.024). Because hand dominance was not assessed, it is unclear whether this side-specific difference reflects true functional asymmetry or differences in dominant hand distribution between age groups. Resilience and self-efficacy scores were slightly higher among older participants, although this potential difference did not reach statistical significance (Table 1).

### 3.3. Differences by Treatment Modalities

Because treatment modalities were self-reported and non-mutually exclusive (participants could receive more than one treatment modality), analyses by treatment category are descriptive and reflect overlapping subgroups rather than independent comparisons. Differences were observed in lower-extremity physical performance (SPPB) by treatment modality, with those who underwent surgery having a mean SPPB score of 10.0 and those who did not having a mean SPPB score of 12. Individuals who received multiple treatments had shorter τPCr (50.8 s ± 14.5) than those who received a single treatment (61.0 s ± 28.3), suggesting shorter phosphocreatine recovery times in this overlapping subgroup. However, those receiving multiple treatment modalities had a longer mean time since initial cancer diagnosis than those receiving only one treatment modality (6.89 ± 4.51 vs. 1.50 ± 0.71). Additionally, the mean age was higher among those receiving multiple treatment modalities than among those receiving only one (55.33 ± 4.40 vs. 44.00 ± 3.00). Participants who received multiple cancer treatments also had lower handgrip strength (Left = 47.49 ± 14.18 lbs., Right = 49.17 ± 14.65 lbs.) but similar self-reported fatigue (52.11 ± 7.23) compared to participants who received single treatments. Participants who received immunotherapy reported higher CRF, longer τPCr, weaker handgrip strength, and lower average steps per day than participants who did not receive immunotherapy (Table 2). These treatment-related patterns should not be interpreted as comparative or causal effects.

### 3.4. Correlations

Physical function assessments, like the Short Physical Performance Battery (SPPB), did not correlate significantly with skeletal muscle mitochondrial oxidative capacity, as measured by τPCr. The τPCr was also not significantly correlated with upper-extremity muscular strength or measures of fatigue and fatigability in the total sample. However, among participants less than 65 years of age, a negative correlation was observed between τPCr and PROMIS-F (ρ = −0.71), such that longer recovery times were associated with lower reported fatigue. In this subgroup, positive correlations were also observed between τPCr and resilience and self-efficacy (Table 3). Subgroup correlations are inherently unstable given the very small sample sizes and should be interpreted cautiously. Because multiple correlations were explored, nominal *p*-values are descriptive and not inferential.

## 4. Discussion

Interpretation of these findings requires substantial caution. This exploratory pilot analysis was conducted in a small overall sample, including very small age- and treatment-based subgroups, and was not powered to detect stable subgroup effects. Accordingly, all observed associations should be considered hypothesis-generating rather than confirmatory, and no causal inferences can be drawn. Potentially important variables, including comorbidities, time since treatment completion, cumulative toxicity burden, and inflammatory markers, were not assessed and may confound the relationships observed. In addition, multiple fatigue instruments were used, each capturing related but distinct constructs. These methodological considerations limit the extent to which biological or clinical interpretations can be inferred from the present data.

Within this exploratory context, we observed patterns suggesting possible relationships among age, cancer treatment modalities, skeletal muscle energetics, physical function, and fatigue. Given the pilot design, interpretation should emphasize descriptive patterns and effect sizes rather than statistical significance. One notable finding was the absence of a significant association between τPCr and physical performance (SPPB scores), indicating that skeletal muscle mitochondrial oxidative capacity may not directly correspond to objective lower-extremity functional performance in this sample. However, among participants younger than 65 years, τPCr was significantly associated with fatigue. Given the very small subgroup size (*n* = 8), this observation should be interpreted conservatively and may not be stable.

Among younger patients, longer τPCr (indicating lower skeletal muscle mitochondrial function) was associated with lower self-reported fatigue. This counterintuitive direction further underscores the need for caution. The association may reflect statistical instability, residual confounding, or heterogeneity across fatigue measures rather than a reproducible physiological relationship. Although younger adults with cancer have been reported to experience greater fatigue burden [28], the present findings are insufficient to support age-specific mechanistic conclusions. The absence of association between τPCr and SPPB appears to suggest that mitochondrial energetics, subjective fatigue, and functional capacity may operate through partially distinct pathways, consistent with the multidimensional nature of fatigue [29].

It is important to distinguish biological capacity, functional performance, and subjective fatigue as related but separable constructs. τPCr derived from ^31^P-MRS reflects skeletal muscle oxidative capacity at the cellular level and indexes mitochondrial recovery kinetics following exertion. In contrast, functional measures such as SPPB scores, handgrip strength, and free-living step counts represent integrated performance influenced by neuromuscular coordination, conditioning, motivation, and environmental context. Subjective fatigue measures capture perceived symptom burden and interference, which are shaped not only by physiological capacity but also by psychological and behavioral factors. The lack of correlation among these domains should not be interpreted as inconsistency, but rather as evidence that biological capacity, functional execution, and symptom perception may be partially decoupled. In small exploratory samples, such decoupling cannot establish a mechanism; however, it highlights the necessity of multidimensional assessment when examining the complex relationships among self-report symptoms, energetics, and physical function.

Age-related differences were observed in mitochondrial and functional measures. Participants aged 65 years and older had longer τPCr than those younger than 65 years, a pattern consistent with lower mitochondrial oxidative capacity. Participants aged 65 years and older also had lower average steps per day and lower upper-extremity strength, although these findings were not statistically significant, likely due to the small sample size. The magnitude of the right-hand grip strength difference was notable; however, hand dominance was not assessed, and this side-specific finding should be interpreted cautiously. These age-related changes are consistent with a prior report describing declines in mitochondrial biogenesis, efficiency, and ATP production capacity with aging [30]. Age-related mitochondrial alterations have been associated with reduced skeletal muscle performance and exercise tolerance. Additionally, age-related losses in lean body mass and muscle quality [31] may contribute to declines in physical capacity during survivorship. These findings offer a preliminary basis for considering skeletal muscle energetics as a factor in age-related physical function disparities in cancer survivors. Whether τPCr-derived MRS can effectively serve as a biomarker for these disparities remains to be confirmed through larger-scale longitudinal studies.

Treatment-related patterns were similarly descriptive. Participants who received multiple treatment modalities had shorter τPCr than those receiving a single modality; however, because treatment modalities overlapped and the times since last treatment were not available, this pattern should be interpreted descriptively rather than comparatively. Because the time since treatment completion was not available in the dataset, the influence of the survivorship phase on symptoms and mitochondrial recovery cannot be evaluated. Recent longitudinal survivorship research demonstrates that fatigue may follow distinct long-term trajectories, with some survivors experiencing persistent or increasing fatigue many years after treatment while others show stable or declining symptoms, suggesting considerable heterogeneity in post-treatment symptom evolution [32,33]. Variation in time since last treatment may therefore meaningfully influence both symptom burden and underlying biological recovery processes. Longitudinal studies that explicitly incorporate treatment timing and extended follow-up are needed to clarify these relationships.

Mitochondrial recovery dynamics may vary according to treatment timing, cumulative toxicity, and post-treatment activity levels. Experimental literature indicates that mitochondrial respiration and bioenergetic efficiency can improve following treatment-related stress, particularly with rehabilitation or increased physical activity [34,35]. Additionally, participants with multiple treatments had lower upper-extremity strength and lower lower-extremity physical performance, suggesting that mitochondrial bioenergetic measures and functional performance may not vary in parallel within this small sample. This pattern is theoretically consistent with the possibility that physiological recovery and physical performance may follow partially distinct trajectories. Such differences could potentially be influenced by factors, unmeasured in the present study, including protein synthesis, muscle regeneration capacity, and neuromuscular coordination [36].

We observed higher fatigue, lower muscle strength, and fewer steps per day among participants undergoing immunotherapy than among those receiving other treatments. These findings are descriptive and should not be interpreted as evidence of treatment-specific effects. Immune checkpoint inhibitors have been associated with systemic inflammatory activation and neuromuscular toxicities such as myositis [37], and inflammatory cytokine signaling has been linked to mitochondrial dysfunction [38] and impairing regeneration [39]. However, inflammatory markers, toxicity grading, and treatment timing were not assessed in this study. Any proposed mechanistic links, therefore, remain speculative and cannot be inferred from the present data.

Similarly, individuals who received multiple treatment modalities had lower handgrip strength, which may reflect the cumulative physical toll of undergoing several treatment modalities; however, overlapping exposures and small subgroup sizes limit interpretability. While combinations such as immunotherapy and chemotherapy can increase therapeutic response, synergistic responses between treatments that increase immune response might also lead to increased fatigue and muscular effects [39]. The current findings do not allow determination of relative contribution or directionality.

### Limitations

Given the small sample size of this analysis, the results are largely exploratory in nature and warrant further investigation. Results should be interpreted cautiously and primarily used to inform effect size estimation for future studies. Additionally, while significant correlations between τPCr and variables such as patient-reported fatigue and self-efficacy were reported in participants under 65 years of age, no significant correlations were observed in participants 65 or older. This is likely due to the older subgroup having only 3 participants, which results in statistical instability and limited interpretability of subgroup findings.

Moreover, participation in the ^31^P-MRS sub-study was optional, and detailed screening or eligibility counts were not systematically documented, raising the possibility of selection bias and limiting the generalizability of findings from this imaging subgroup. In addition, several key clinical covariates, including time since last treatment, cancer stage, and comorbid conditions, were not available in this secondary dataset. The absence of these variables threatens internal validity, as both self-selection into imaging and unmeasured clinical heterogeneity may independently influence mitochondrial function, fatigue, physical performance, and psychological outcomes, thereby introducing unmeasured confounding.

Another limitation is the use of consumer-grade wearable devices to capture daily step counts. Although these devices have shown acceptable validity in clinical populations, they may underestimate certain activity patterns (e.g., slow walking, upper-limb movements) that are common among cancer survivors, potentially leading to conservative estimates of physical activity. Furthermore, the absence of formal wear-time validation criteria means that incomplete or inconsistent device wear may have further contributed to the underestimation of daily step counts. Such underestimation would be expected to bias activity estimates toward lower values and attenuate associations between physical activity and mitochondrial or symptom-related measures.

## 5. Conclusions

These findings collectively highlight the multifactorial nature of how biological, physiological and psychological factors are affected by age and cancer treatment modalities to shape cancer health outcomes. The study findings underscore the need for personalized approaches to optimize survivorship outcomes. Additionally, this study provides effect size estimates for the relationship between treatment and patient-reported outcomes with measures of skeletal muscle mitochondrial energetics. Future research should seek to characterize the relationship of mitochondrial energetics with age, fatigue, and treatment in larger cohorts. These cohorts should have stratified sampling by age to ensure adequate age-specific sample sizes and have a standardized characterization of the treatment exposure, including dose and time since treatment. Specifically, the effect of immunotherapy and multiple concurrent therapies with energetics warrants further investigation, as do potential interaction effects of age on the relationship between mitochondrial energetics and fatigue. Understanding these relationships can inform the development of more targeted interventions to improve long-term health trajectories of cancer survivors.

## Figures and Tables

**Table 1:** Patient-Reported Outcomes, Physical Function, and Skeletal Muscle Energetics by Age Among Cancer Survivors (*n* = 11).

Category	Variables	<65 Years (*n* = 8)	≥65 Years (*n* = 3)	*p*	Cohen’s d
Phosphocreatine recovery time (τPCr), s		50.06 ± 16.55	59.49 ± 16.54	0.307	−0.50
Self-report fatigue And fatigability	PROMIS-F (T-score)	50.03 ± 12.90	55.77 ± 8.56	0.352	−0.41
	FACIT-F total score	115.22 ± 31.71	102.22 ± 27.64	0.307	0.37
	Physical fatigability	21.38 ± 18.70	25.50 ± 26.16	0.889	−0.17
	Mental fatigability	17.88 ± 13.07	7.00 ± 5.66	0.400	0.81
Lower-extremity physical performance	SPPB	10.75 ± 2.05	9.67 ± 2.08	0.503	0.46
Upper-extremity muscular strength (lbs)	Handgrip strength (Left)	54.29 ± 13.86	35.22 ± 7.50	0.102	1.32
	Handgrip strength (Right)	56.29 ± 13.19	36.43 ± 6.86	0.024 *	1.45
Average steps per day (×10^3^)		7.48 ± 3.92	5.58 ± 1.83	0.57	0.47
Resilience		30.12 ± 4.67	34.00 ± 1.73	0.099	−0.82
Self-efficacy		96.63 ± 22.52	95.00 ± 13.12	0.838	0.07

Note: Values are presented as mean ± standard deviation. Group comparisons were conducted using independent samples *t*-tests and are exploratory in nature; results should not be considered confirmatory. Cohen’s d was calculated as (<65 − ≥65) and is provided for descriptive magnitude estimation only, given the small and imbalanced subgroup sizes. PROMIS-F = Patient-Reported Outcomes Measurement Information System Fatigue; FACIT-F = Functional Assessment of Chronic Illness Therapy–Fatigue; SPPB = Short Physical Performance Battery; τPCr = phosphocreatine recovery time constant. Asterisks indicate *p* < 0.05 (unadjusted). Average steps per day (×10^3^) = mean daily step count in thousands of steps (e.g., 6.5 = 6500 steps/day).

**Table 2:** Treatment Differences in Patient-Reported Outcomes, Physical Function, and Skeletal Muscle Energetics Among Cancer Survivors (*n* = 11), mean (standard deviation).

Variables Category		Surgery		Chemotherapy		Radiation		Hormonal Therapy		Immunotherapy		More than 1 Treatment	
		Yes *n* = 7	No *n* = 4	Yes *n* = 5	No *n* = 6	Yes *n* = 6	No *n* = 5	Yes *n* = 3	No *n* = 8	Yes *n* = 6	No *n* = 5	Yes *n* = 9	No *n* = 2
**Phosphocreatine recovery time (τPCr), s**		52.2 (16.2)	53.4 (19.1)	53.7 (16.3)	51.8 (17.8)	52.9 (17.5)	52.3 (16.8)	54.3 (14.0)	52.0 (18.0)	56.8 (18.3)	47.7 (13.8)	50.8 (14.5)	61.0 (28.3)
**Self-reported** **fatigue**	PROMIS-F (T-score)	51.5 (10.2)	51.7 (15.9)	54.7 (6.07)	49.2 (14.6)	52.5 (8.4)	52.7 (15.5)	41.9 (7.3)	55.2 (11.3)	56.2 (13.8)	48.3 (7.1)	52.1 (7.2)	54.8 (29.8)
	FACIT-F total score	116.5 (23.4)	112.3 (41.0)	107.7 (24.5)	115.0 (35.8)	110.0 (26.3)	113.8 (36.8)	136.7 (12.1)	102.3 (29.4)	103.8 (34.7)	121.2 (23.0)	112.5 (23.7)	108.0 (66.5)
**Self-reported** **fatigability**	Physical	23.0 (14.0)	21.0 (26.9)	23.5 (15.9)	21.3 (21.9)	19.6 (10.2)	24.8 (25.9)	15.3 (10.7)	25.1 (21.3)	25.6 (25.5)	18.8 (10.6)	20.3 (13.1)	30.0 (42.4)
	Mental	16.2 (10.0)	15.0 (17.5)	15.3 (10.7)	16.0 (14.6)	18.0 (10.8)	13.4 (15.0)	14.3 (14.0)	16.3 (13.0)	14.2 (15.2)	17.2 (10.8)	14.9 (9.8)	19.0 (26.9)
**Lower-extremity physical** **performance**		10.0 (2.0)	12.0 (0.0)	10.6 (2.0)	10.5 (2.1)	10.0 (2.3)	11.2 (1.3)	10.7 (1.5)	10.5 (2.1)	10.8 (1.8)	10.2 (2.17)	10.2 (2.0)	12.0 (0.0)
**Upper-extremity strength**	Left	47.1 (15.9)	52.2 (14.7)	42.2 (13.6)	54.3 (14.7)	48.2 (12.0)	50.0 (19.5)	55.5 (13.8)	46.5 (15.5)	45.4 (15.8)	53.3 (14.4)	47.5 (14.2)	56.0 (22.5)
	Right	50.0 (16.6)	52.5 (13.0)	40.4 (21.2)	56.3 (13.7)	49.6 (10.9)	48.4 (26.6)	57.8 (15.3)	45.8 (19.4)	46.3 (14.3)	56.4 (14.7)	49.17 (14.7)	58.6 (17.7)
**Average steps per day (×10^3^)**		7.1 (4.5)	6.6 (9.5)	6.19 (1.5)	7.7 (4.8)	5.5 (1.4)	8.3 (4.4)	11.6 (6.1)	5.7 (1.3)	5.9 (1.3)	8.4 (5.3)	7.0 (3.9)	6.7 (0.3)
**Resilience**		30.0 (5.4)	31.8 (1.9)	31.8 (3.7)	30.7 (5.2)	31.0 (5.6)	31.4 (3.1)	30.3 (7.5)	31.5 (3.3)	32.6 (2.3)	29.4 (5.9)	30.9 (4.8)	32.5 (0.7)
**Self-efficacy**		113.7 (17.4)	99.3 (23.9)	108.6 (13.9)	109.7 (25.0)	109.3 (25.8)	109.0 (12.1)	126.3 (15.6)	102.8 (17.7)	104.2 (20.6)	115.2 (19.0)	107.8 (20.9)	115.5 (17.7)

Note: Values are presented as mean (standard deviation). Treatment modalities were self-reported and are non-mutually exclusive; participants may be represented in more than one category. These profiles are descriptive and reflect overlapping subgroups rather than independent between-group comparisons. PROMIS-F = Patient-Reported Outcomes Measurement Information System Fatigue; FACIT-F = Functional Assessment of Chronic Illness Therapy–Fatigue; τPCr = phosphocreatine recovery time constant. Hand grip strength reported in pounds (lbs.). Average steps per day (×10^3^) = mean daily step count in thousands of steps.

**Table 3:** Spearman’s nonparametric correlations with τPCr.

		Spearman’s Rho (ρ), (95% Confidence Interval)		
		Total Sample (*n* = 11)	<65 Years (*n* = 8)	≥65 Years (*n* = 3)
**Self-report fatigue**	PROMIS-F	−0.40	−0.71 *	0.50
	FACIT-F	0.38	0.62	−0.50
**Self-report fatigability**	Physical	−0.39	−0.29	−1.0
	Mental	−0.50	−0.28	−1.0
**Lower-extremity physical performance: SPPB score**		−0.19	−0.14	−0.50
**Upper-extremity muscular strength: hand grip strength (lbs)**	Left	−0.53	−0.57	0.50
	Right	−0.52	−0.57	0.50
**Average steps per day (×10^3^)**		−0.04	0.11	−0.50
**Resilience**		0.61 *	0.38	0.87
**Self-efficacy**		0.74 **	0.76 *	0.50

Note: PROMIS-F = Patient-Reported Outcomes Measurement Information System Fatigue; FACIT-F = Functional Assessment of Chronic Illness Therapy–Fatigue; SPPB = Short Physical Performance Battery; τPCr = phosphocreatine recovery time constant. * *p* < 0.05 (exploratory, unadjusted). ** *p* < 0.01 (exploratory, unadjusted). Subgroup correlations (≥65, *n* = 3) are not interpretable beyond effect-size illustration.

## Data Availability

The data used in this study are available from the corresponding authors upon request.
